# V_2_CT_X_ MXene-based hybrid sensor with high selectivity and ppb-level detection for acetone at room temperature

**DOI:** 10.1038/s41598-023-30002-6

**Published:** 2023-02-22

**Authors:** Sanjit Manohar Majhi, Ashraf Ali, Yaser E. Greish, Hesham F. El-Maghraby, Saleh T. Mahmoud

**Affiliations:** 1 Department of Physics, College of Science, United Arab Emirates University, 15551 Al-Ain, United Arab Emirates; 2 Department of Chemistry, College of Science, United Arab Emirates University, 15551 Al-Ain, United Arab Emirates; 3 Department of Ceramics, National Research Center, NRC, Cairo, 12622 Egypt

**Keywords:** Nanoscience and technology, Nanoscale devices, Nanoscale materials, Materials for devices

## Abstract

High-performance, room temperature-based novel sensing materials are one of the frontier research topics in the gas sensing field, and MXenes, a family of emerging 2D layered materials, has gained widespread attention due to their distinctive properties. In this work, we propose a chemiresistive gas sensor made from V_2_CT_x_ MXene-derived, urchin-like V_2_O_5_ hybrid materials (V_2_C/V_2_O_5_ MXene) for gas sensing applications at room temperature. The as-prepared sensor exhibited high performance when used as the sensing material for acetone detection at room temperature. Furthermore, the V_2_C/V_2_O_5_ MXene-based sensor exhibited a higher response (S% = 11.9%) toward 15 ppm acetone than pristine multilayer V_2_CT_x_ MXenes (S% = 4.6%). Additionally, the composite sensor demonstrated a low detection level at ppb levels (250 ppb) at room temperature, as well as high selectivity among different interfering gases, fast response-recovery time, good repeatability with minimal amplitude fluctuation, and excellent long-term stability. These improved sensing properties can be attributed to the possible formation of H-bonds in multilayer V_2_C MXenes, the synergistic effect of the newly formed composite of urchin-like V_2_C/V_2_O_5_ MXene sensor, and high charge carrier transport at the interface of V_2_O_5_ and V_2_C MXene.

## Introduction

With the growing awareness of rapid environmental pollution and the importance of health diagnoses, designing smart, sensitive sensors has become a frontier research topic in the gas sensing field^1^. The development of the Internet of Things (IoT) has enabled the integration of several types of active sensors into a single network, allowing users to be warned of impending risk through smart technologies^2^. One category of sensors, gas sensors (a sub-class of chemical sensors), has played a pivotal role in monitoring hazardous gases and volatile organic compounds (VOCs) in industries, indoor areas, and medical environments to improve the safety and security of humans^3–5^. Another category, point-of-care smart sensing devices, has garnered attention for achieving real-time diagnoses of diseases^6^. For example, human breath is a mixture of various gases, such as N_2_, O_2_, CO_2_, water vapor, trace amounts of VOCs (acetone, ammonia, isoprene, etc.), and inorganic gases (H_2_S, CO, NO, etc.). These gases are generated either endogenously (in the body) or exogenously (from environmental contaminants)^7,8^. In particular, acetone is a useful biomarker for diagnosing diabetes; it is a byproduct of the metabolic process of ketosis and is expelled from the body via waste or breath^9^. Concentrations of acetone range from 0.2–0.9 parts per million (ppm) in healthy individuals and 0.9–1.8 ppm in diabetic patients^10^. Local statistics indicated that nearly 17.3% of the United Arab Emirates (UAE) population aged 20–80 had type 2 diabetes in 2017, while nearly 1 million people had type 1 diabetes, ranking the country fifteenth worldwide^11^. Compared to a conventional blood glucose test, which can be painful, exhale breath analysis is a promising, non-invasive, non-hazardous, and cost-effective approach to detect acetone^12,13^. Therefore, novel, high-performance sensing materials are needed for designing sensitive gas sensor devices to detect acetone in the breath. Novel materials have been employed to detect VOCs and toxic gases, including metal-oxide semiconductor (MOXS)-based chemiresistors^14^, carbon nanotubes (CNTs)^10^, and graphene-based, two-dimensional (2D) materials^15^. However, although MOXS gas sensors are frequently employed as effective transducer gas sensors, their high working temperature is a significant practical obstacle^3^. Room temperature (RT)-operated gas sensing has been targeted as a solution to this challenge. While CNTs and graphene-based 2D materials can operate at RT, their sluggish reaction and low response behaviors impede practical applications^16,17^. Therefore, alternative sensing materials that can operate at RT and show enhanced sensing properties are necessary.

MXenes are a novel class of emergent 2D transition-metal carbides/nitrides that are typically synthesized by selectively etching Al from the MAX phase^18^. MXenes have demonstrated their potential in myriad applications, including gas sensors, due to their unusual features, such as their surface functional groups, versatile chemistry, exceptional solubility, high metallic conductivity, and high specific surface area^17^. Since the discovery of Ti_3_C_2_T_x_ MXene, numerous studies on other MXene materials and their properties have been conducted^19,20^. Ti_3_C_2_T_x_ MXene has been widely used for gas sensors because of its high stability, metallic behavior, and easy synthesis^21^. Vanadium-based MXenes, such as V_2_CT_x_, also hold potential for gas sensor applications, and a few recent studies have reported on their gas sensing properties^22–24^. Furthermore, a series of reports have examined the fabrication of MXene-based composites^25–27^ for improving the efficiency of gas sensors. Moreover, MXene can be directly converted to metal oxides at different thermal conditions^28,29^.

To our knowledge, no research has been conducted on the synthesis of V_2_O_x_ from thermally annealed V_2_CT_x_ MXene for gas sensing. Considering the above-stated advantages of MXene-based composite materials, we hereby propose a novel strategy for using thermally oxidized, multilayered V_2_CT_x_ MXene to synthesize V_2_CT_x_ MXene-derived, urchin-like vanadium oxide (V_2_O_x_) hybrid structures for gas sensing applications. We used a hydrothermal method to synthesize multilayered V_2_CT_x_ MXenes. These pristine V_2_CT_x_ MXene materials were transformed into urchin-like vanadium oxide (V_2_O_x_) hybrid structures at different annealing temperatures, and then we investigated the acetone-sensing properties at RT. Compared to the pristine V_2_CT_x_ MXene, the urchin-like vanadium oxide (V_2_O_x_) hybrid sensor showed improved acetone-sensing performances at ppb-level detection with high selectivity, long-term stability, and good repeatability when utilized as a sensing material. The acetone sensing properties and potential sensing mechanisms are discussed in this manuscript.

## Experimental part

### Synthesis of multilayer V_2_CT_x_ MXene

The synthesis approach used in this study involved mixing 1.5 g of LiF (99.995%, Sigma Aldrich) powder with 30 ml of HCl (37% GR, Sigma Aldrich) in a 100-ml PTFE bottle by stirring. Subsequently, 1.5 g of V_2_AlC MAX-phase powders (≥ 90% purity, APS: ≤ 40 µm, American Elements, USA) were slowly dropped into the above LiF-HCl solution, and the mixture was stirred for 10 min to achieve complete mixing. This mixture was then sealed in a Teflon-lined steel autoclave and heated at 90 ℃ for 5 days in an oven. Upon completion of the reaction, the etched solution was washed and centrifuged with copious amounts of DI water until the pH of the solution reached ~ 6. A detailed description of the washing procedure is shown in Supplementary Fig.1. The resultant V_2_CT_x_ MXene precipitates were dried at 80 °C for 12 h in a vacuum oven.

### Synthesis of V_2_CT_x_-derived composites

The as-obtained V_2_CT_x_ powder was calcined at different temperatures ranging from 300–450 °C at a rate of 1 °C/min. The composition and morphology of the calcined powders were investigated.

### Material characterization

The prepared MXene samples were characterized using powder X-ray diffraction (PXRD; Rigaku-600-C, USA) with a CuKα X-ray (λ = 1.5406) at a scan rate of 1°/min. Scanning and transmission electron microscopies (SEM; Thermo scientific, Quattro S, and TEM; Tecnai Spirit G2, Netherlands) were used to examine the microstructure of the solid materials. Additionally, an energy-dispersive X-ray attachment was used to evaluate the elemental composition of the prepared materials. X-ray photoelectron spectroscopy (XPS) analysis was also performed using the PHI5000 Version Probe III. Thermogravimetric (TG) and differential thermogravimetric analysis (DTA) of the V_2_CT_x_ MXene sample was conducted using the METTLER Toledo TGA2 STAR^e^ System. The UV–Visible Diffuse Reflectance Spectra (DRS) was measured for V_2_O_5_ MXene using Shimadzu UV-3600 diffuse reflectance spectrophotometer (200–800 nm, BaSO_4_ is used to record the baseline).

### Preparation of sensing device and gas-sensing measurement

The sensing device was built on a 1 × 1 cm alumina (Al_2_O_3_) substrate with Pt-interdigitated electrodes (IDEs) (Supplementary Fig. 2). Then, the sensor device was coated with a paste made from 10 mg of the MXene sample and 10 μL of α-terpineol. This paste was applied via screen printing followed by drying in an oven at 80 °C for 12 h. The sensor was evaluated using a Teflon-based gas-sensing chamber (Supplementary Fig. 2(C)). A test gas was mixed with synthetic air as a carrier gas and was passed into the sealed testing chamber through Bronkhorst mass flow controls (MFCs). The change in electrical resistance or current signals for different concentrations of analytes were recorded using a source meter (Keithley, KI 236) with 1 V of bias voltage. A LABVIEW program was used to record the readable signal data from the interface between the KI 236 source meter and the MFCs. Different gases, including CO, H_2_, H_2_S, acetone, ethylene, and CO_2_, were used for selectivity testing. Air and N_2_ were used as carrier and flushing gases, respectively. The effect of humidity on gas sensors was also studied by exposing the sensor to different humid conditions (0–90%), which were measured by a commercially procured humidity meter operated at RT (23 °C).

## Results and discussions

### Morphological and structural characterization

**Figure 1:**
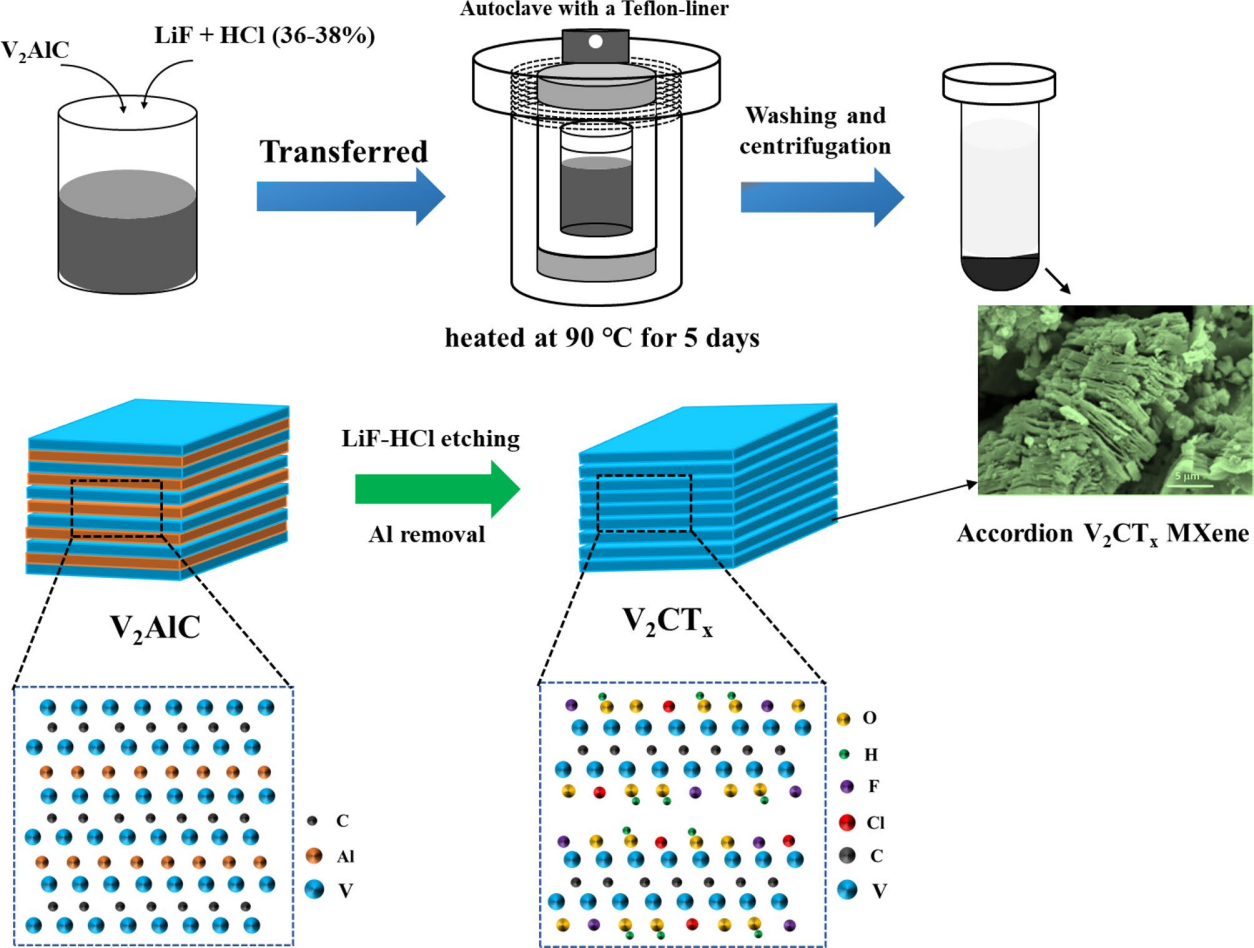
Schematic illustration of the synthesis process of accordion-like V_2_CT_x_ MXene from V_2_AlC MAX-phase powder.

**Figure 2:**
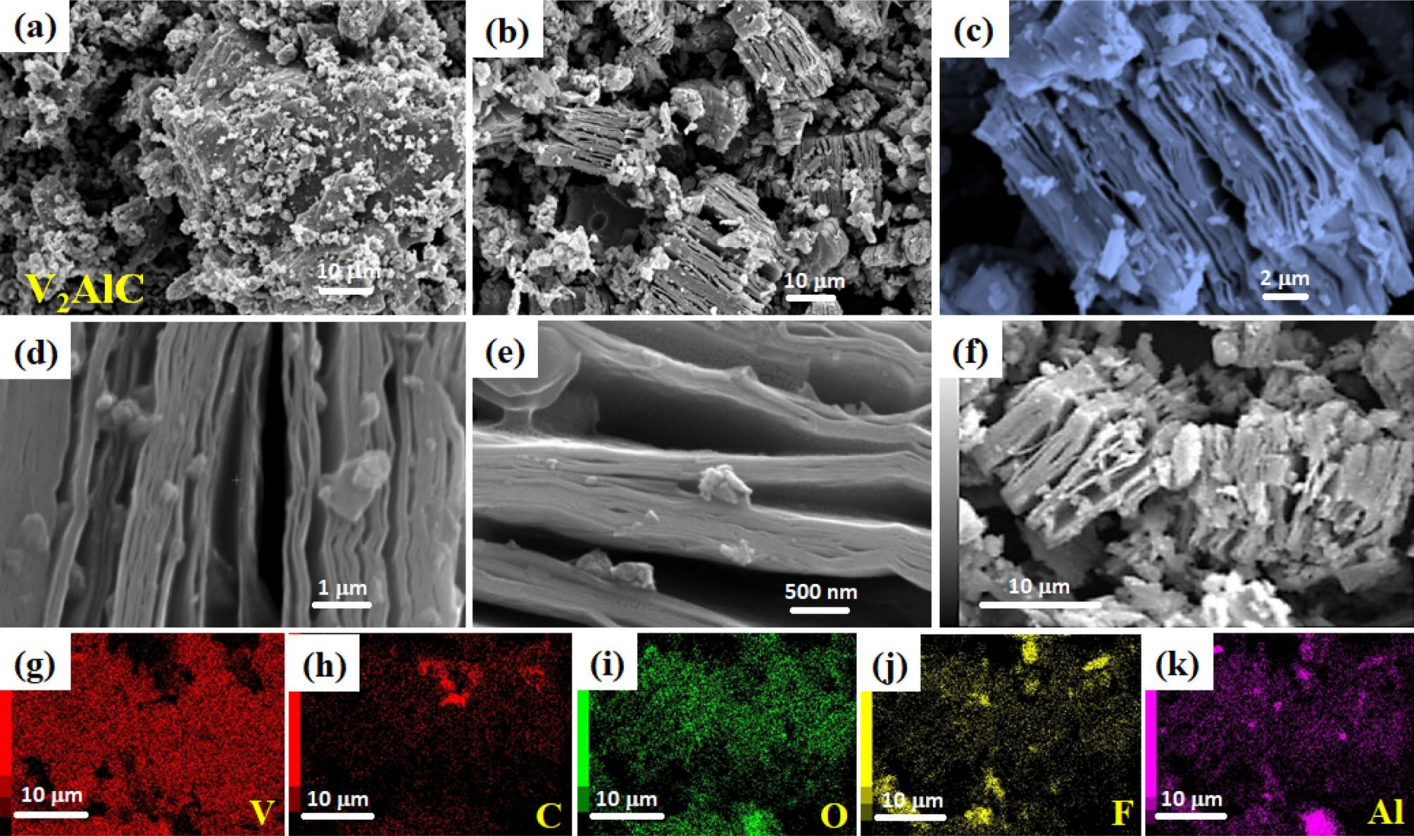
SEM images of V_2_AlC MAX-phase powders (**a**), V_2_CT_x_ MXene (**b–f**), and EDX-Mapping analysis of V_2_CT_x_ MXene samples (**g–k**) taken from (**f**) image.

**Figure 3:**
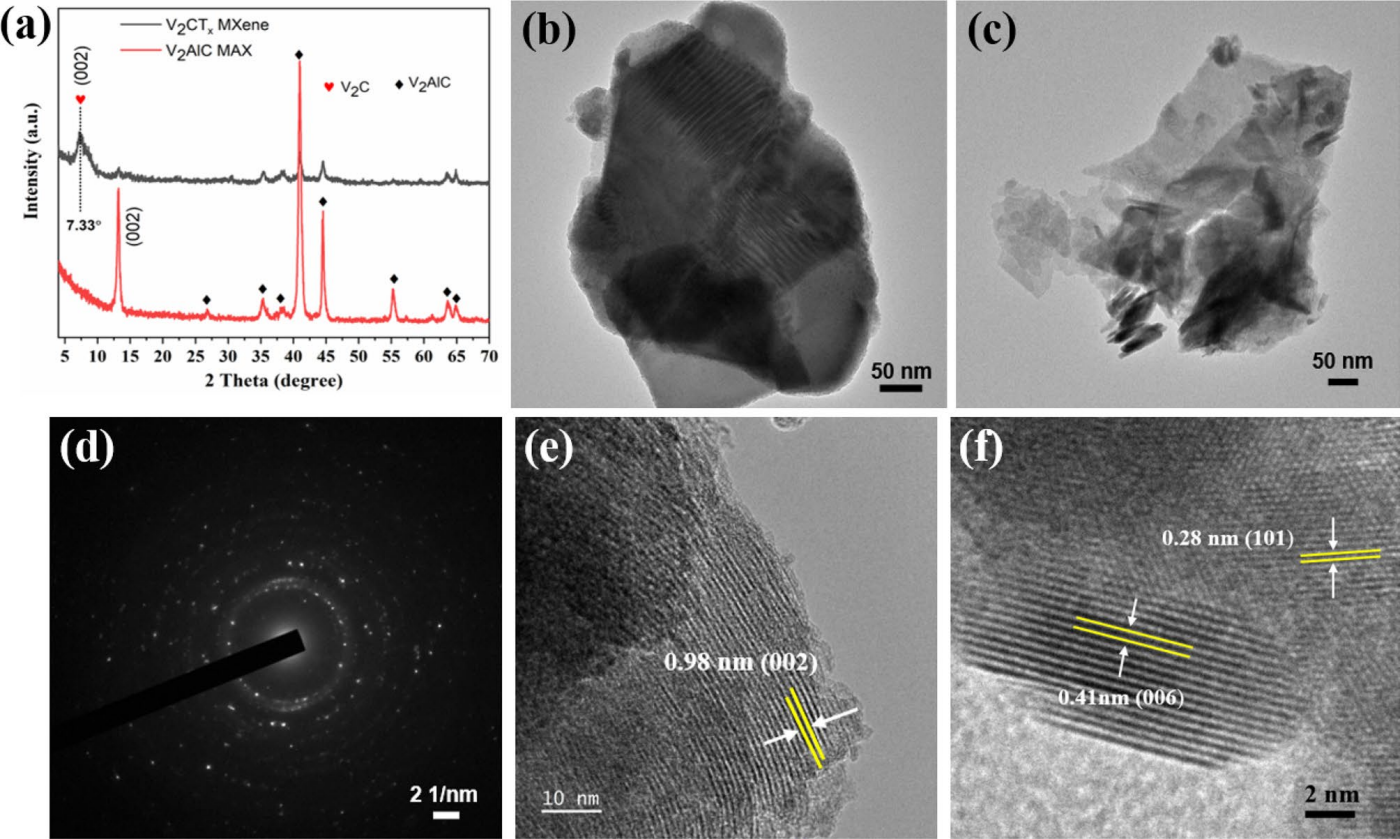
XRD pattern of V_2_AlC MAX-phase powders versus V_2_CT_x_ MXene (**a**), TEM images of V_2_CT_x_ MXenes (**b**, **c**), SAED pattern (**d**), and (**e**, **f**) HRTEM image of V_2_CT_x_ MXene.

The V_2_CT_x_ MXenes were synthesized by a hydrothermal method using LiF and HCl solution at 90 °C for 5 days, as shown in Fig. 1. The morphologies of the V_2_AlC MAX phase and its exfoliated product, V_2_CT_x_ MXene materials, were characterized by SEM and TEM, respectively (as shown in Figs. 2 and 3). Figure 2a depicts the SEM image of the V_2_AlC MAX phase materials. As seen in the figure, the surface of the V_2_AlC MAX phase material comprised typical dense particles with no layer structures, while tiny particles (1–10 μm) were present on its surface. After the hydrothermal treatment with LiF-HCl etchant, typical accordion-like V_2_CT_x_ MXenes with a few or multiple layers were formed (Fig. 2b–f). In Fig. 3d–e, high-magnification SEM images depict how Al-etching made interlayer spacings more apparent, with a gap of a few nanometers between the V_2_CT_x_ MXene sheets. The particle size of the V_2_CT_x_ MXene ranged from 1–20 μm, and the individual MXene sheets were connected. The elemental analysis of the as-prepared V_2_CT_x_ MXene was also examined using EDX-Mapping analysis (Fig. 2g–k), by which the presence of C, V, F, and O was confirmed. The presence of a low-intensity Al signal may have been due to the continued presence of V_2_AlC, which was not etched.

The XRD patterns of the V_2_AlC MAX phase and the pristine V_2_CT_x_ MXene were also investigated, as shown in Fig. 3a. All diffraction peaks (shown in the red pattern) corresponded to the V_2_AlC phase (JCPDS, No. 29-0101)^24^. No other phase peaks were found in the pattern, indicating the high purity of the prepared V_2_AlC phase. The intensities of all peaks representing the MAX phase V_2_AlC decreased in the XRD patterns of the V_2_CT_x_ phase, confirming the elimination of Al layers from V_2_AlC. Meanwhile, a broad peak appeared at a low angle (2θ = 7.33°), corresponding to the (002) plane of as obtained accordion-like V_2_CT_x_ MXene^24^. The formation of the peak indicated the formation of MXene sheets.

To investigate the morphology of the V_2_CT_x_ MXene further, we performed TEM analysis as shown in Fig. 3b–c. It shows that 2D flake structures are stacked in a few single layers. Figure 3d shows the SAED pattern of V_2_CT_x_ MXene, which demonstrated the polycrystalline nature of the prepared materials. Figure 3e-f shows the corresponding high-resolution TEM images of V_2_CT_x_ MXene. Figure 3e shows an inter-planar spacing of 0.98 nm (002), while Fig. 3f shows lattice-fringes of 0.41 nm and 0.28 nm corresponding to (006) and (101) planes of V_2_CT_x_ MXene.

**Figure 4:**
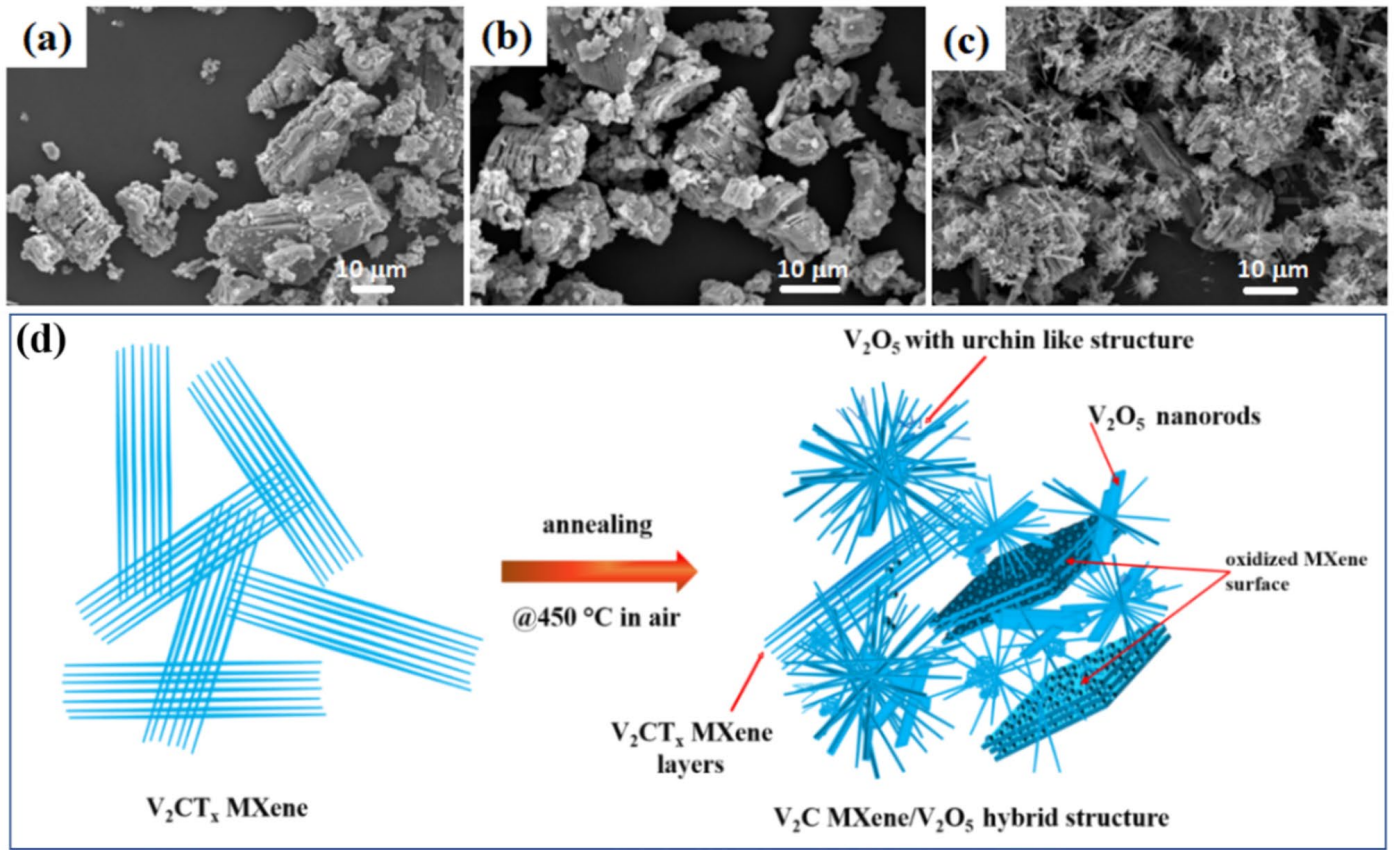
SEM images of V_2_CT_x_ MXene calcined at different temperatures: 300 °C (**a**), 350 °C (**b**), 450 °C (**c**), and (**d**) Schematic diagram of the formation of the V_2_C MXene-derived, urchin-like V_2_O_5_ structure annealed at 450 °C in air.

The prepared V_2_CT_x_ MXene materials were annealed at different temperatures to produce MXene-derived hybrid structures and evaluate their sensing properties. The V_2_CT_x_ MXene samples were calcined at 300, 350, and 450 °C in air. Hereafter, these samples are designated as V_2_C-300, V_2_C-350, and V_2_C-450, respectively. Morphological and structural analyses of the calcined V_2_C materials were also conducted using SEM and XRD. The SEM micrographs shown in Fig. 4 indicated that calcination temperatures of 300 and 350 °C did not affect the multilayer structure of the MXene, where the particle size of the V_2_CT_x_ MXene ranged from 1–50 μm (Fig. 4 a–b). However, at an annealing temperature of 450 °C, the layer structure of the V_2_C-450 MXene material was transformed into typical urchin-like microstructures (Fig. 4c). Numerous spike-like threads and several micro rods joined together to form the urchin-type or flower-type morphologies. The schematic diagram of this structure is shown in Fig. 4d. We also studied the effect of annealing temperature on the elemental analysis of the V_2_CT_x_ MXene materials (Supplementary Tables 1–3). Based on the data presented in Tables S1–S3, oxygen levels were slightly increased with increasing annealing temperature, with the highest concentration at an annealing temperature of 450 °C.

**Figure 5:**
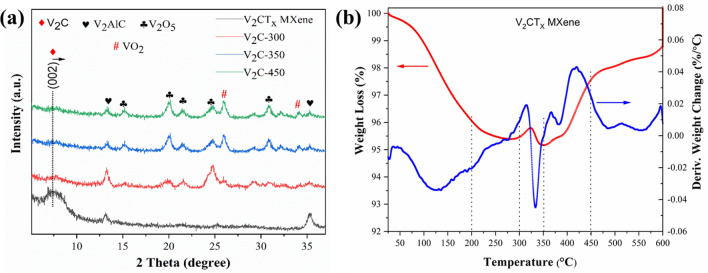
(**a**) XRD patterns of pristine V_2_CT_x_ MXene and MXene-derived V_2_C-300–450 samples calcined at different temperatures (from 300–450 °C); (**b**) TG–DTA analysis of the V_2_CT_x_ MXene sample.

To examine the phase composition of the thermally annealed V_2_CT_x_ MXene, we performed XRD analysis (Fig. 5a). We found that the V_2_C MXene peak (002) was shifted to a higher theta angle as the annealing temperature increased; these findings supported our previous results^30^. However, compared to that of pristine V_2_CT_x_ MXene, the intensity of the (002) peak was decreased in the annealed samples, which may have been due to the formation of V_2_O_x_ phases. When the annealing temperature increased to 450 °C, the V_2_CT_x_ MXene was oxidized to urchin-like interconnected networks of V_2_O_5_ inherited from the V_2_CT_x_ MXene. Henceforth, the V_2_CT_x_ MXene-derived V_2_C-450 sample will be designated as urchin-like V_2_CT_x_/V_2_O_5_ MXene. The XRD pattern of V_2_C-450 corresponded to the diffraction peaks of orthorhombic V_2_O_5_ (JCPDS no. 41-1426)^31^. Other impurities of the V_2_AlC phase (JCPDS, No. 29-0101) were evident in the XRD pattern^24^. The partial oxidation of the V_2_CT_x_ MXene was further shown using TGA-DTG analysis (Fig. 5b). A weight loss of 3.4% was observed at 120 °C, which was due to the evaporation of the physically adsorbed, interlayer water molecules^32,33^. However, this weight loss was followed by a minor weight gain at 330 °C, which indicated the onset of oxidation of the V_2_CT_x_ MXene. This finding agreed with the XRD results of the calcined V_2_CT_x_ MXene (Fig. 5a). Two events of weight gain were also observed at 370 and 420 °C, with a more pronounced weight gain at 420 °C. These events correspond to the partial oxidation of the V_2_CT_x_ MXene at this temperature, as observed in the XRD patterns of the corresponding samples (Fig. 5a). The oxidation of the V_2_CT_x_ MXene was likely due to the dissociation of hydroxyl surface terminations (-OH/-O/-F) and the interaction of V_2_CT_x_ MXene with O_2_ molecules from the air during calcination, which resulted in V_2_O_x_^34^. However, the partial oxidation of V_2_CT_x_ MXene ceased at 600 °C, after which weight gain was no longer observed.

### XPS analysis

**Figure 6:**
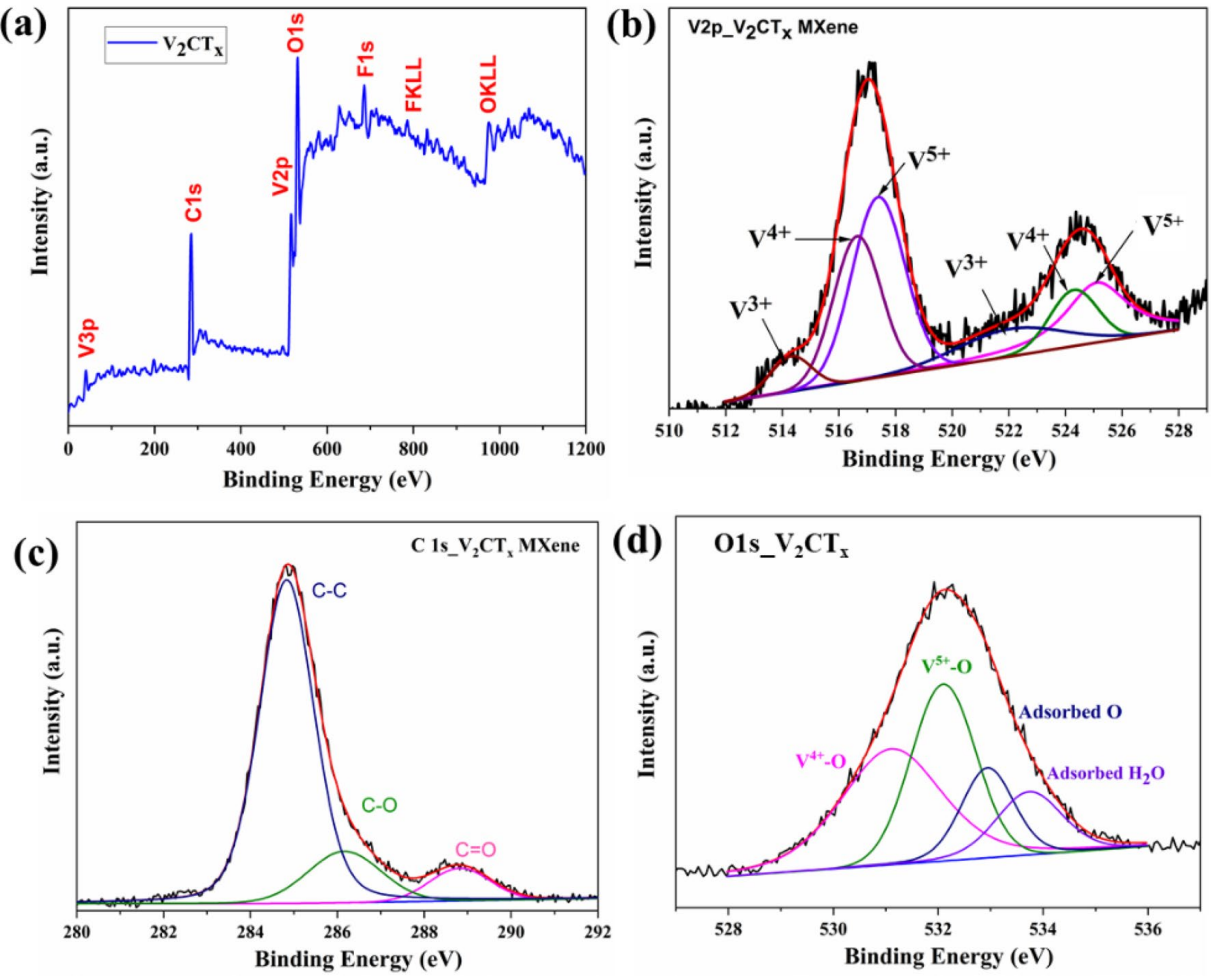
XPS analysis of V_2_CT_x_ MXene: (**a**) total survey, (**b**) V_2p_, (**c**) C1s, and (**d**) O1s spectra.

After etching or delamination, MXene surfaces are spontaneously re-occupied with different functional groups, such as OH, O, and F^35^. Thus, to investigate the chemical states in the V_2_CT_x_ MXene further, we performed XPS analysis, as shown in Fig. 6. This analysis confirmed that the V_2_CT_x_ MXene surface was occupied with V, O, C, and F elements. The synthesis method we utilized played a pivotal role in determining the specific quantities of these groups^23^. Figure 6a shows the total survey in the whole range, whereas Fig. 6b–d depicts the XPS spectra of V_2p_, C1s, and O1s of V_2_CT_x_ MXene. The high-resolution spectrum of V_2_p revealed the presence of vanadium, predominately in its V^5+^ and V^4+^ forms. The V_2p_ spectra could be fitted by six peaks for V^5+^ at 517.39 and 525.11 eV, for V^4+^ at 516.66 and 524.30 eV, and for V^3+^ at 514.27 and 522.16 eV^36,37^. Of these peaks, the peak area of the V^5+^ oxidation state was higher than the peaks of other oxidation states. Vanadium was mainly present in the high-valence state of V^4+^ and V^5+^, which was likely due to the existence of a combination of monolayer oxide/vanadium oxide on the V_2_CT_x_ MXene surface^23,38–40^. The C1s spectrum (Fig. 6c) was fitted to three peaks at 288.84, 286.16, and 284.84 eV, which could be assigned to C=O, C–O, and C–C, respectively^38,41^. Finally, the O1s spectra (Fig. 6d) could be fitted to four peaks at 533.75, 532.93, 532.09, and 531.1 eV, which could be assigned to the presence of adsorbed water, adsorbed O, V^5+^–O, and V^4+^–O, respectively. The above components could be ascribed to different oxygen and hydroxyl-containing functional groups, vanadium oxide (VO_x_), and intercalated water resulting from the partial surface oxidation of V_2_CT_x_ MXene^37,42^.

XPS analysis was also performed to determine the surface properties of the annealed samples of the V_2_CT_x_ MXene material (Supplementary Fig. 3). Supplementary Fig. 3a shows the V_2p_ spectrum of V_2_CT_x_/V_2_O_5_ MXene, which was Gaussian-fit with two 2p doublets of vanadium corresponding to two oxidation states: V^5+^ at 517.51 and 525.11 eV (major peaks) and V^4+^ at 516.51 and 524.23 eV (minor peaks). The intensity of the V_2p_-V^5+^ peak was higher than that of the pristine, non-annealed V_2_CT_x_ MXene. The O1s XPS spectra of V_2_CT_x_/V_2_O_5_ MXene (Supplementary Fig. 3b) displayed four fitting peaks at 529.92, 530.56, 531.81, and 532.73 eV, which corresponded to V^4+^–O, V^5+^–O, adsorbed O, and adsorbed water, respectively. Moreover, a broad peak was observed at 531.81 eV for adsorbed O in the V_2_C-450 MXene sample. The V^3+^ peak disappeared when the V_2_CT_x_ MXene was annealed at 450 °C.

### Acetone-sensing performance of V_2_CTx MXene-based sensors

**Figure 7:**
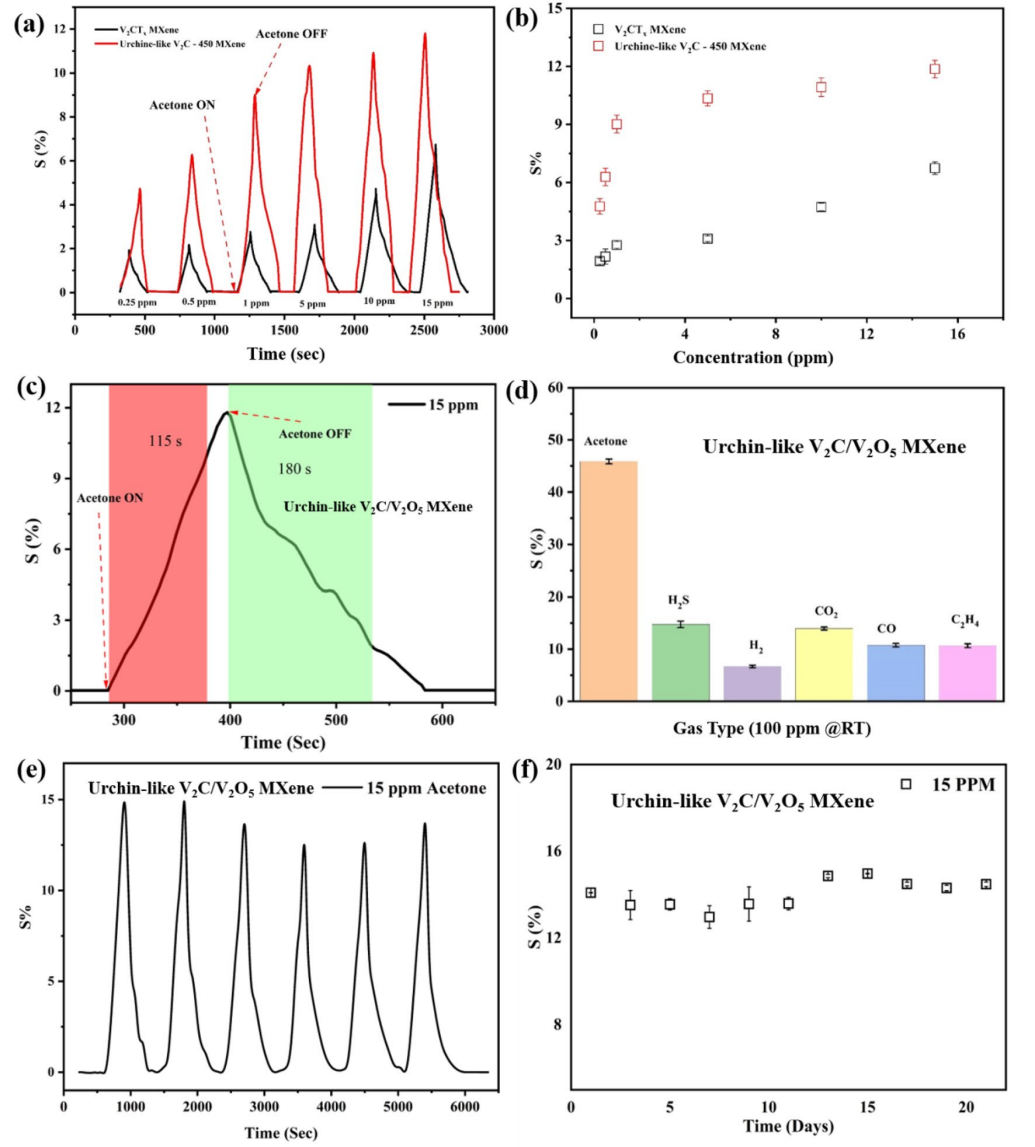
(**a**, **b**) Response transients of V_2_CT_x_ MXene and urchin-like V_2_CT_x_/V_2_O_5_ MXene sensors toward acetone vapor (0.25–15 ppm) tested at RT; (**c**) responses of pristine V_2_CT_x_ MXene and urchin-like V_2_CT_x_/V_2_O_5_ MXene sensors at different acetone vapor concentrations; (**c**) response-recovery plot of urchin-like V_2_CT_x_/V_2_O_5_ MXene sensor; (**d**) selectivity test of urchin-like V_2_CT_x_/V_2_O_5_ MXene sensor for 100 ppm of different gases; (**e**) repeatability and (**f**) long-term stability (21 days) tests for urchin-like V_2_CT_x_/V_2_O_5_ MXene sensor toward 15 ppm acetone at RT (23 °C).

We evaluated our prepared sensor devices, which were based on pristine V_2_CT_x_ MXene and urchin-like V_2_CT_x_/V_2_O_5_ MXene, for their acetone-sensing performance at RT (23 °C). Figure 7(a) illustrates the response /recovery plot of the pristine V_2_CT_x_ MXene and urchin-like V_2_CT_x_/V_2_O_5_ MXene sensors when tested with acetone vapor (0.25–15 ppm). Compared to conventional metal-oxide-based chemiresistors, MXene shows a positive response (an increase of resistance)^43^, likely due to its metallic characteristics. We observed a significant increase in the amplitudes of the urchin-like V_2_CT_x_/V_2_O_5_ MXene sensor compared to pristine V_2_CT_x_ MXene. Supplementary Fig. 4 displays the dynamic resistance variations of pristine V_2_CT_x_ and urchin-like V_2_CT_x_/V_2_O_5_ MXene sensors when tested with acetone vapor (0.25–15 ppm).

Both the pristine V_2_CT_x_ MXene and urchin-like V_2_CT_x_/V_2_O_5_ MXene sensors showed positive response behaviors. The initial resistance of the urchin-like V_2_CT_x_/V_2_O_5_ MXene sensor was higher than that of the pristine V_2_CT_x_ MXene^25^. We attributed the lower baseline resistance of V_2_CT_x_ MXene to its excellent metallic properties and intrinsic high electrical conductivity. Our findings verified that V_2_CT_x_ is the most important part of electrical transportation systems and that V_2_O_5_ is a contributor to an improved sensing response. During the gas-sensing experiment, we exposed the sensor device to the target gas for 120 s, followed by purging with synthetic air for 300 s to remove any remaining gas molecules. We used Eq. (1) to calculate the response (S (%)) of the sensors:

1 $$S \left(\%\right)= \frac{{R}_{g}-{R}_{a}}{{R}_{a}} \times 100$$

where R_a_ and R_g_ are resistances when the sensor is exposed to air and target analytes, respectively. Figure 7b describes the responses of the pristine V_2_CT_x_ MXene and urchin-like V_2_CT_x_/V_2_O_5_ MXene sensors as a function of the acetone vapor concentrations. Our results showed that when the acetone vapor concentration was raised from 0.25 to 15 ppm, the response values of the V_2_CT_x_ MXene sensor also increased. The V_2_CT_x_ MXene sensor showed a 6.7% response (S%) to 15-ppm acetone vapor, while the urchin-like V_2_CT_x_/V_2_O_5_ MXene sensor showed a response of 11.9%, with a low detection limit of 4.76% for 0.25 ppm. The response time and recovery time of a gas sensor are critical parameters for determining how quickly the sensor responds to the target gases. A gas sensor’s response time (τ_response_) is the time needed for 90% changes in resistance during exposure to the target gas, whereas the recovery time (τ_recovery_) is the time required for a 90% return to baseline resistance after the target gas is switched off and synthetic air is turned on. Figure 7c illustrates the response and recovery performance of the urchin-like V_2_CT_x_/V_2_O_5_ MXene sensor toward 15 ppm acetone. The response and recovery times of this sensor toward 15 ppm acetone were 115 s and 180 s, respectively. Selectivity is also an essential factor for a gas sensor in terms of practical application; it is the sensor’s ability to distinguish a target gas from other interfering gases. The selectivity test of the urchin-like V_2_CT_x_/V_2_O_5_ MXene sensor is shown in Fig. 7d. In addition to acetone, different gases were chosen for this test, including H_2_, CO, H_2_S, CO, and C_2_H_4_. The urchin-like V_2_CT_x_/V_2_O_5_ MXene sensor demonstrated the highest response of 47% for 100 ppm acetone, displaying high selectivity against acetone vapor.

Repeatability is another important sensor parameter. As shown in Fig. 7e, the urchin-like V_2_CT_x_/V_2_O_5_ MXene sensor was exposed to six consecutive cycles of acetone vapor (100 ppm) at RT (23 °C). The results indicated good repeatability, with negligible variations in resistance. The stability of a gas sensor is another important parameter for practical applications. To examine stability, the urchin-like V_2_CT_x_/V_2_O_5_ MXene sensor was tested for three weeks (Fig. 7f). The response barely fluctuated, indicating good long-term stability of the fabricated sensor. The effect of relative humidity (RH) on gas sensors is an important study in the gas sensing field. The urchin-like V_2_CT_x_/V_2_O_5_ MXene sensor was tested toward 100 ppm acetone and evaluated its effect on RH at RT. As shown in Supplementary Fig. 6, at 50% of the RH environment, the response value changed from 46 to 20%. However, with a further increase in the RH% up to 90%, the response value decreases rapidly indicating that the V_2_CT_x_/V_2_O_5_ MXene sensor shows a poor response in high humidity. To assess the gas-detecting properties of the as-prepared sensors, we compared our findings with those of other research (see Table 1). As shown, our prepared urchin-like V_2_CT_x_/V_2_O_5_ MXene-based hybrid sensor showed promising acetone sensing.

**Table 1:** A summary of recent studies on various acetone-sensing-based chemiresistive gas sensors.

Materials	Synthesis method	Response (S or S%)	Response time/Recovery time	Conc. (ppm)	Temp. (℃)	References
Li-V_2_CT_x_	LiF-HCl etching	−0.6%	NA	500	RT	^22^
Na-V_2_CT_x_	NaF-HCl etching	−1%	NA	500	RT	^22^
HF-V_2_CT_x_	HF etching	−1%	NA	500	RT	^22^
In_2_O_3_/Ti_3_C_2_T_x_	HF-etching	2	6.5/3.5 s	5	RT	^26^
Mo_2_CT_x_ MXene	HF-etching	0.14	NA	140	RT	^44^
Ti_3_C_2_T_x_/SnO/SnO_2_	HF-etching	12.1^#^	18/9 s	100	RT	^45^
Ti_3_C_2_T_x_/SnO MXene	HF-etching	1.44	NA	200	RT	^25^
MXene/CuO/rGO	HF-etching	52.09	6.5/7.5 s	100	RT	^46^
Ti_3_C_2_T_x_ MXene NFs	LiF-HCl etching	1.50%	90 /102 s	10	RT	^47^
rGO/In_2_O_3_	Hydrothermal	40*	NA	20	RT	^48^
Ti_3_C_2_T_x_-F Mxene	LiF-HCl etching	3.5	39/139 s	30	RT	^49^
PEDOT: PSS/MXene	LiF-HCl etching	3.4%^##^	NA	100	RT	^50^
Au@Co_3_O_4_ core–shell NPs	In-situ self-assembly method	27.5^#^	233 s/280 s	10	250	^51^
W_18_O_49_/Ti_3_C_2_T_x_	HF etching	11.6	5.6/6 s	20	300	^52^
V_4_C_3_T_x_	HF etching	2.65%	40 s/–	100	RT	^53^
Ag-Ti_3_C_2_T_x_	HF etching	0.5%	NA	10	RT	^54^
Ti_3_C_2_T_x_/WSe_2_	HF-etching	−4.5%	NA	40	RT	^55^
2D d-V_2_CT_x_ nanosheets	HF etched	0.0226	NA	100	RT	^23^
Urchin-like V_2_CT_x_/V_2_O_5_ MXene	LiF-HCl etching	11.9%	115/180 s	15	23 °C	This work

*S = R_a_/R_g_, ^#^S = R_g_/R_a_, S% = (R_g_−R_a_)/R_g_, ^##^S = (R_a_−R_g_)/R_a_.

### Gas-sensing mechanism

**Figure 8:**
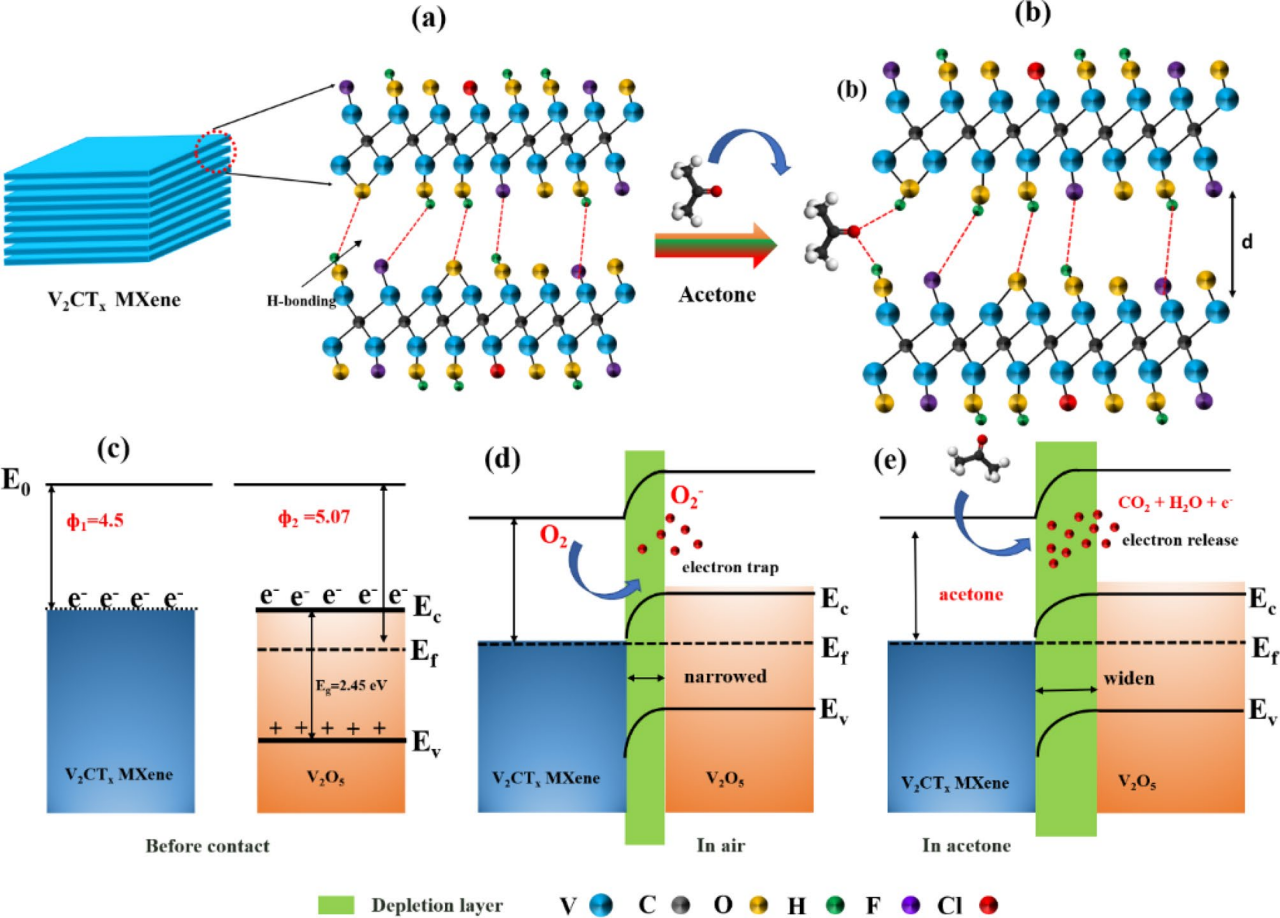
(**a**, **b**) Sensing mechanism of pristine V_2_CT_x_ MXene toward acetone at RT; energy-band diagram of the V_2_CT_x_ − V_2_O_5_ heterostructure before contact (**c**), in the air (**d**), and in acetone (**e**).

The gas-sensing properties of the prepared sensors, V_2_CT_x_ MXene and urchin-like V_2_CT_x_/V_2_O_5_ MXene, were tested at RT. Among them, the urchin-like V_2_CT_x_/V_2_O_5_ MXene sensor demonstrated high sensitivity to acetone vapor. The high sensing performances of urchin-like V_2_CT_x_/V_2_O_5_ MXene can be explained by two possible sensing mechanisms, based on the potential formation of H-bonding and the possible synergistic effect of the V_2_CT_x_/V_2_O_5_ MXene. Let’s first start with the discussion on the acetone sensing mechanism of pristine V_2_CT_x_ MXene. As evident from the previous research that most of MXene materials exhibit metallic characteristics and p-type sensing behaviors^22,23,44,45,47^. The surface of V_2_CT_x_ MXene is covered with functional groups of –O, –OH, and –F, as confirmed by XPS (Fig. 6), and these groups form different hydrogen bonds^56^ with each other, as depicted in Fig. 8a. Additionally, the pristine V_2_CT_x_ MXene comprises an accordion structure with multilayers, and interconnected nanosheets with different functional groups, which maintain the flow of charge carriers throughout the V_2_CT_x_ MXene, and thus high-conductivity. This results in the low baseline resistance of the sensor and a p-type sensing behavior is observed, which can be seen from the response transient graph in Fig. 7c. When the target gas-like acetone molecules are exposed to the MXene, it forms bonds on the MXene surface with the different functional groups (Fig. 8b). This phenomenon contributes to the hindering of the conducting channel in MXene, thereby increasing the resistance of the sensor. Therefore, the response transient graphs show the increase in resistance when the acetone molecules are exposed to the V_2_CT_x_ MXene sensor. Additionally, due to their large molecular size compared to other gases, when acetone gas molecules enter into the multilayer sheets of MXene, the contact performance of the MXene will be hindered due to the steric effect (Fig. 8b)^53,57^. As a result, the resistance of the V_2_CT_x_ MXene sensor varies and hence the response of the sensor.

On the other hand, the sensor based on V_2_CT_x_/V_2_O_5_ MXene heterostructures displayed an enhanced response compared to the pristine V_2_CT_x_ MXene-based sensor. In particular, the resistance of the urchin-like V_2_CT_x_/V_2_O_5_ MXene sensor was higher than that of the pristine V_2_CT_x_ MXene (as shown in Supplementary Fig. 2), which may be due to the Schottky barrier formation^58^. The V_2_CT_x_/V_2_O_5_ MXene heterostructures-based sensor caused a positive response toward acetone similar to the pristine V_2_CT_x_ MXene. Yao et *al*. observed similar sensing behavior for Ti_3_C_2_T_x_/SnO composites^25^; in their study, the sensor showed a negative response towards ammonia and a positive response towards acetone. Possible reactions at the interface between V_2_CT_x_ and V_2_O_5_ can provide insights into the sensing mechanism for explaining the enhanced acetone sensing response of the urchin-like V_2_CT_x_/V_2_O_5_ MXene sensor. The energy level and band structures of the urchin-like V_2_CT_x_/V_2_O_5_ MXene sensor before and after contacts are illustrated in Fig. 8c-e, in which the work functions (ф) are 4.5 eV^56^ and 5.07eV^59^ for V_2_CT_x_ and V_2_O_5_, respectively. The band gap energy of V_2_O_5_ was calculated to be 2.45 eV from the Tauc plot calculated from the UV–Visible DRS (as shown in Supplementary Fig. 5a, b). The Fermi energy difference between V_2_CT_x_ and V_2_O_5_ triggers the charge transport process at the interface contact. The electrons will flow from V_2_CT_x_ Mxene to V_2_O_5_ until the fermi levels reach equilibrium, which results in band bending and depletion layer formation. Typically, the O_2_ molecules in the air that adsorbed into the surface of urchin-like V_2_O_5_ MXene sensor trap electrons from the interface to form oxygen ionic species (O_2_^−^)^55^. This narrows down the depletion layer and triggers the movement of the charge carrier at the interface and results in high conductivity in the system, as shown in Fig. 8d. Thus, the sensing mechanism can be explained by the dominant nature of the electron concentration, which was likely related to the suppression of charge-carrier recombination; hence, low charge carriers resulted in lower resistance^45,60^. Upon exposure to the acetone vapor, the pre-adsorbed oxygen species react with acetone molecules to release trapped electrons (Fig. 8e) widening the depletion layer, and is reflected in the increases of sensor resistance^58^. Being a composite of a 2D material and typical MOXs, the V_2_CT_x_/V_2_O_5_ MXene heterostructures sensor displayed a synergistic effect that was strongly related to the improvement of the sensor response. More specifically, the heterostructures made by the numerous urchin-like nanosized V_2_O_5_ rods that are spread on the micron-sized V_2_CT_x_ MXene can facilitate the adsorption efficiency of acetone gas molecules due to their possible high active sites and surface exposure^58^. During the adsorption of acetone molecules, the metallic nature of V_2_CT_x_ may be compensated for the low conductivity of urchin-V_2_O_5_ rods, resulting in a faster electron exchange rate, which in turn led to faster response/recovery times and increased sensing response^45,61^.

## Conclusions

In summary, accordion-type vanadium carbide (V_2_CT_x_) MXene was successfully fabricated using a one-step hydrothermal synthesis technique at 90 °C. Multilayered V_2_CT_x_ MXenes were partially transformed into urchin-type V_2_O_5_ structures (V_2_CT_x_/V_2_O_5_ MXene) at 450 °C calcination temperature. The morphological, structural, and surface properties of both materials were investigated, and both sensors were evaluated for their efficiency in sensing acetone at RT. The dominant metallic characteristics of the V_2_CT_x_ MXene were reflected in a positive response toward acetone. The as prepared V_2_CT_x_ MXene-derived, urchin-like V_2_CT_x_/V_2_O_5_ MXene hybrid sensor showed an improved response (S% = 11.9) toward 15 ppm acetone at RT compared to pristine V_2_CT_x_ MXene sensor. The sensor demonstrated a ppb-level detection with a low limit of detection (250 ppb). Furthermore, the V_2_CT_x_/V_2_O_5_ MXene sensor exhibited high selectivity to acetone among different interfering gases, fast response-recovery time (115s/180s), and excellent reproducibility and long-term stability (21 days) at RT.

## Supplementary Information

Supplementary Information.

## Data Availability

The experimental analyzed data in this study can be available from the corresponding authors on request.
