# Supplementary material for: V2CTX MXene-based hybrid sensor with high selectivity and ppb-level detection for acetone at room temperature
Source: Sci Rep. 2023 Feb 22;13:3114. doi: 10.1038/s41598-023-30002-6 (PMC9947003; doi:10.1038/s41598-023-30002-6)
Supplement: Supplementary file 1 — Supplementary Information. [file 41598_2023_30002_MOESM1_ESM.pdf]

Supplementary Information

V_2CT_X MXene-Based Hybrid Sensor with High Selectivity and ppb-level Detection for Acetone at Room Temperature

Sanjit Manohar Majhi,[1] Ashraf Ali,[1] Yaser E. Greish,[2,3], Hesham F. El-Maghraby,[2,3] Saleh T. Mahmoud[1]*

[1]Department of Physics, College of Science, United Arab Emirates University, Al-Ain 15551, United Arab Emirates

[2]Department of Chemistry, College of Science, United Arab Emirates University, Al-Ain 15551, United Arab Emirates

[3] Department of Ceramics, National Research Center, NRC, Cairo 12622, Egypt

*Corresponding Author

E-mail address: saleh.thaker@uaeu.ac.ae (S. T. Mahmoud)

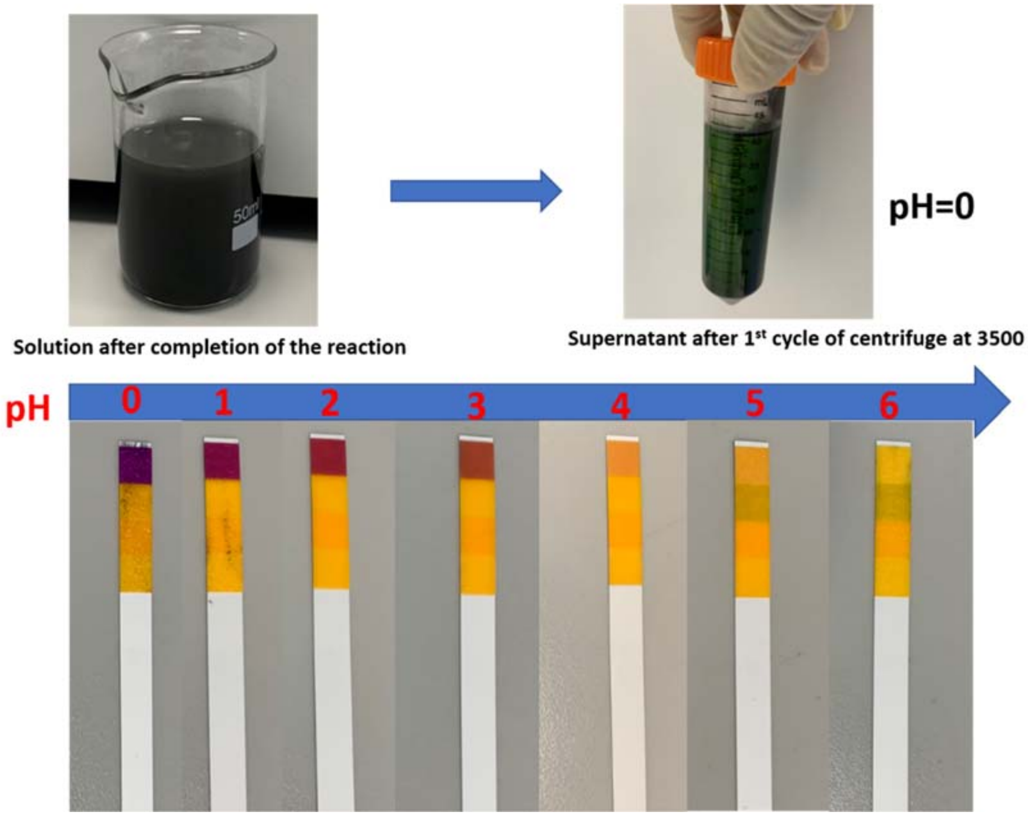

Supplementary Fig. 1. Photographs showing the washing procedure to change the pH of the as-synthesized V_2CT_x MXene sample.

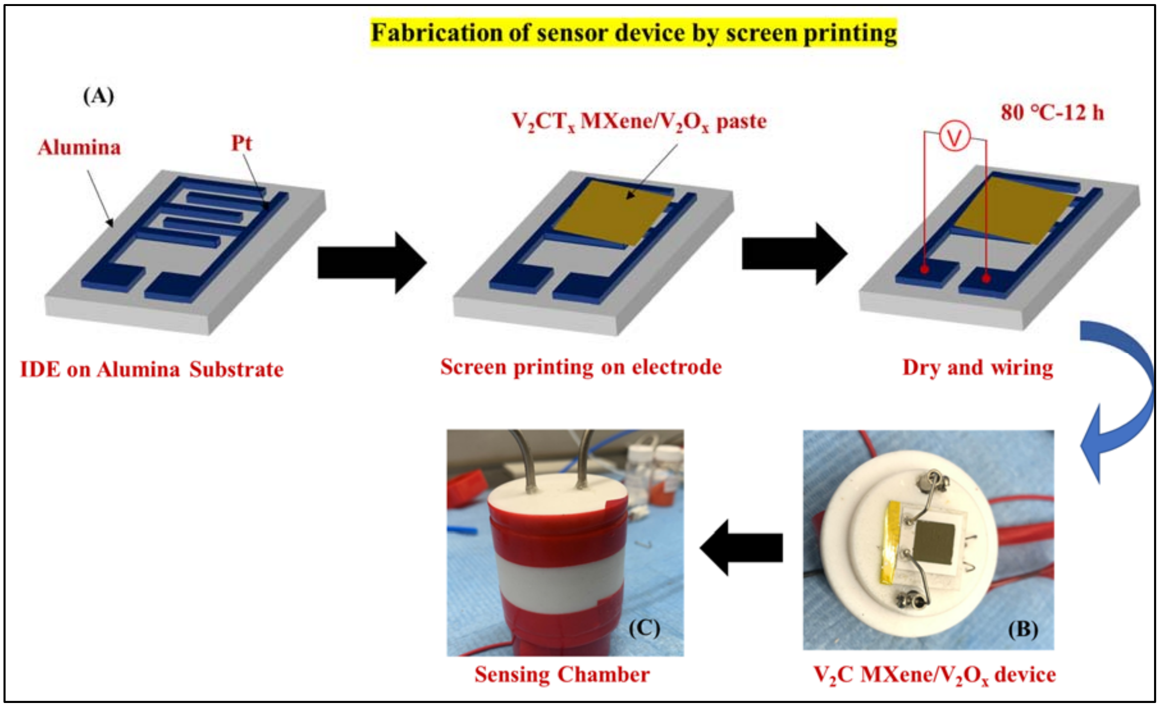

Supplementary Fig.2 (A) Schematic diagram of the sensor device fabrication, (C) sensor device electrical connection setup inside the test chamber; and (D) picture of the sensing laboratory-made Teflon-based gas sensing chamber.

Supplementary Table-1

V_2C-300 MXene Sample

Formula	Mass%	Atom %
**C**	25.08	43.03
**O**	21.34	27.49
**F**	11.47	12.44
**V**	42.12	17.04
**Total**	100.00	100.00

Supplementary Table-2

V_2C-350 MXene Sample

Formula	Mass%	Atom %
**C**	19.13	36.25
**O**	20.05	28.52
**F**	10.74	12.87
**V**	50.07	22.37
**Total**	100.00	100.00

Supplementary Table-3

V_2C-450 MXene Sample

Formula	Mass%	Atom %
**C**	13.67	29.20
**O**	24.76	39.72
**F**	0.07	0.10
**V**	61.50	30.98
**Total**	100.00	100.00

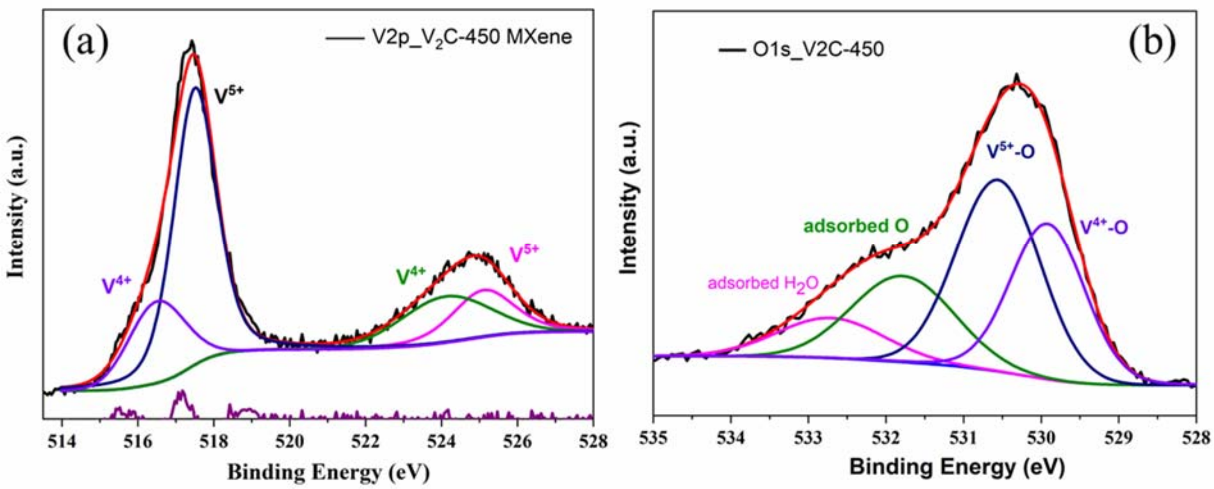

Supplementary Fig. 3. The XPS analysis of V_2CT_x MXene derived V_2C-450 MXene (V_2C/V_2O_5 MXene) (a) V2p spectrum, and (b) O1s spectrum.

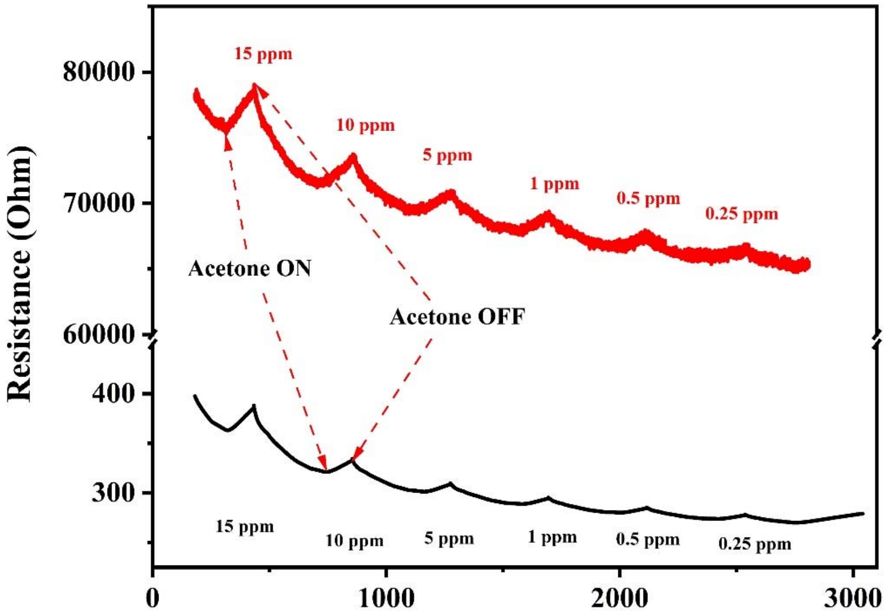

Supplementary Fig. 4. Resistance variations of V_2CT_x Mxene (black line) and V_2C/V_2O_5 MXene (red line) sensors towards acetone vapor (0.25-15 ppm) tested at RT.

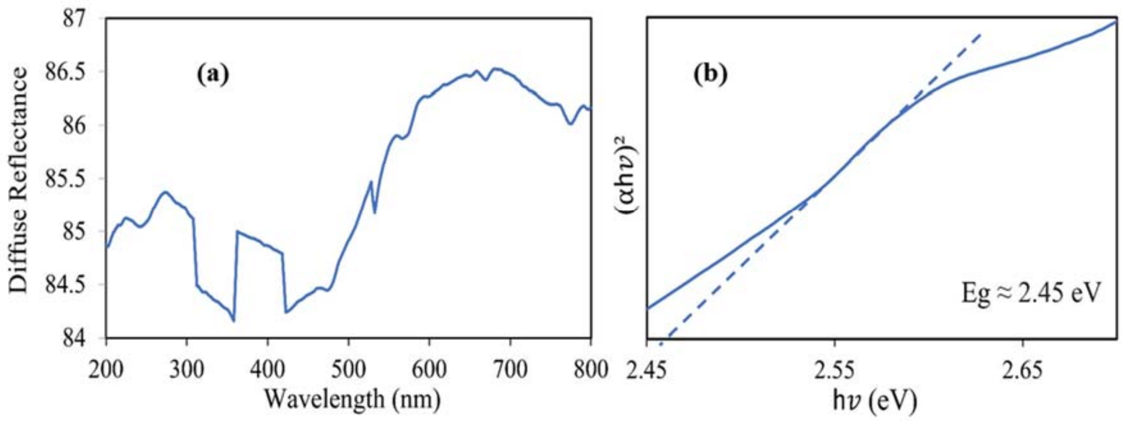

Supplementary Fig. 5 UV-Visible Diffuse Reflectance Spectra of V_2O_5 MXene sample (a), and Tauc plot showing the energy band gap (b).

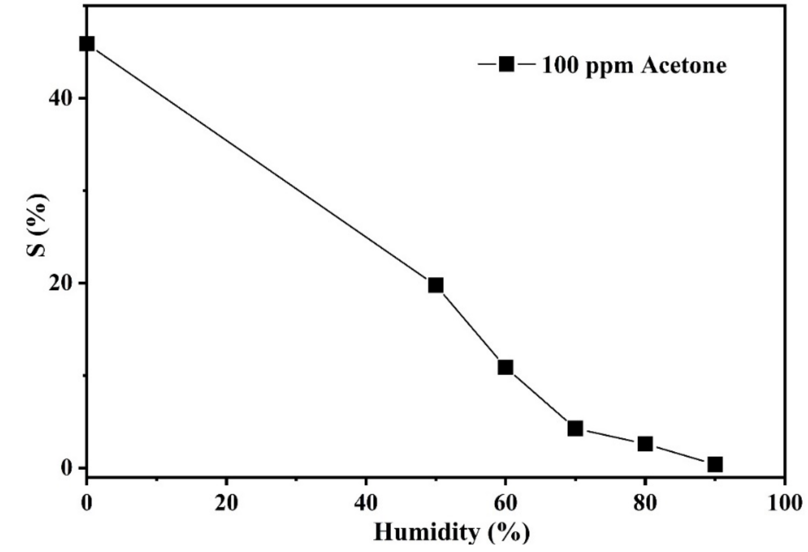

Supplementary Fig. 6 The effect of RH (5 to 90%) on the sensing properties of V_2CT_x/V_2O_5 MXene sensor toward 100 ppm acetone at RT.
